# Advances in Coccygectomy: A Comprehensive Review Evaluating Surgical Techniques for Coccygodynia

**DOI:** 10.3390/brainsci15020213

**Published:** 2025-02-19

**Authors:** Barnabas Obeng-Gyasi, Ethan D. L. Brown, Anoop Sai Chinthala, Gordon Mao

**Affiliations:** 1 Department of Neurological Surgery, Indiana University School of Medicine, 355 W. 16th Street, Goodman Hall Suite 5100, Indianapolis, IN 46202, USA; bobenggy@iu.edu (B.O.-G.); aschinth@iu.edu (A.S.C.); 2 Department of Neurological Surgery, Donald and Barbara Zucker School of Medicine, 500 Hofstra Blvd, Hempstead, NY 11549, USA; ebrown35@northwell.edu

**Keywords:** coccygectomy, coccygodynia, minimally invasive techniques, pain management, surgical approaches

## Abstract

Background: Coccygodynia presents significant challenges in diagnosis and treatment. While coccygectomy has emerged as a crucial intervention for refractory cases, significant heterogeneity exists in surgical techniques. Traditional approaches are increasingly complemented by novel methods, necessitating a comprehensive review of current surgical options. Methods: A comprehensive literature review was conducted using Ovid MEDLINE, Cochrane Library and Embase databases from inception to present. Search terms included “coccygectomy”, “coccydynia”, “coccygodynia”, “coccyx pain” and “tailbone pain”. We analyzed peer-reviewed studies focusing on surgical techniques, outcomes and complications of coccygectomy. Studies were excluded if non-peer-reviewed, non-English without translation, or not directly addressing surgical management. Results: Traditional midline approaches, while common, demonstrate increased wound complications compared to paramedian techniques. Minimally invasive methods, including coccygeoplasty and endoscopic coccygectomy, show promising early outcomes with reduced recovery times. Both partial and complete resections provide significant pain relief, with complete resection potentially offering superior results in severe cases. Wound closure technique significantly impacts surgical success. Conclusions: Optimal outcomes in coccygectomy require individualized surgical approaches incorporating modern techniques like paramedian incision and advanced wound closure. Emerging minimally invasive procedures may further reduce complications and enhance recovery. Treatment success depends on careful patient selection and surgical technique optimization.

## 1. Introduction

Coccygodynia presents significant diagnostic and therapeutic challenges in contemporary spine surgery through its characteristic pain in the coccygeal region [1]. The condition demonstrates a notable gender disparity with higher prevalence in females due to childbirth-related trauma and anatomical variations in pelvic structure [1,2]. The female coccyx maintains a more vertical orientation as an evolutionary adaptation that enlarges the pelvic outlet for childbirth, thus increasing susceptibility to trauma and irritation during routine activities like sitting [3,4]. Obesity represents another key risk factor by altering sitting biomechanics and increasing mechanical loads on the coccyx [5,6]. Most patients present in middle age with symptoms ranging from mild to severe pain alongside inconclusive initial imaging and abnormal coccygeal mobility [7].

Surgical intervention through coccygectomy has emerged as a crucial treatment modality when conservative measures fail [8]. The procedure’s anatomical location presents unique challenges with elevated risks of infection and wound dehiscence that significantly influence surgical approach selection and postoperative outcomes. Recent years have seen substantial evolution in surgical techniques from traditional approaches to innovative minimally invasive methods as surgeons work to optimize patient outcomes [8]. These advancements have expanded the surgical options available while highlighting the importance of careful technique selection based on individual patient factors.

This review provides a comprehensive analysis of contemporary surgical techniques for coccygodynia treatment with particular emphasis on emerging minimally invasive approaches. Through examination of current surgical interventions including both traditional methods and technological innovations, we aim to offer evidence-based insights that guide surgical decision-making. Our analysis evaluates the efficacy, safety and appropriate patient selection criteria across various surgical approaches to enhance treatment outcomes in coccygectomy procedures.

## 2. Methods

### 2.1. Literature Search Strategy

This review synthesizes existing knowledge on surgical techniques used in coccygectomy for treating coccygodynia, emphasizing traditional, minimally invasive and innovative approaches. A comprehensive literature search was conducted through Ovid MEDLINE, Cochrane Library and Embase databases from inception to present with assistance from a medical librarian following the SANRA guidelines for narrative reviews [9]. Key search terms included “coccygectomy”, “coccydynia”, “coccygodynia”, “coccyx pain” and “tailbone pain”. Additional terms were used to capture specific surgical techniques: “minimally invasive”, “endoscopic”, “paramedian” and “surgical outcomes”. Boolean operators combined these terms to optimize search sensitivity and specificity.

### 2.2. Inclusion Criteria

We included peer-reviewed original research, review papers and technical papers focused on surgical interventions for coccygodynia that detailed treatment outcomes and surgical techniques. Case series with five or more patients, comparative studies, systematic reviews and meta-analyses were prioritized. Technical papers describing novel surgical approaches were included regardless of patient numbers to ensure comprehensive coverage of emerging techniques. Articles were limited to English language or those with available English translations. Case reports were considered only if they described novel surgical techniques or unique complications.

### 2.3. Data Extraction and Analysis

Studies were excluded if they were non-peer-reviewed, did not directly address coccygectomy or coccygodynia, focused solely on conservative management, or lacked detailed surgical methodology. One reviewer extracted data systematically using a standardized form capturing: study design, patient demographics, surgical techniques, outcome measures (pain scores, patient satisfaction, disability indices), complications and follow-up duration. We assessed the quality of the included studies by ensuring that surgical studies adhered to the Modified Coleman Methodology Score and systematic reviews followed the PRISMA guidelines. Given the high methodological heterogeneity across studies, we applied a narrative approach to evidence synthesis to provide a comprehensive overview of surgical advancements and clinical implications in coccygectomy.

## 3. Surgical Approaches

Table 1 highlights the surgical techniques that will be discussed in the following section. We provide key findings and discuss their outcomes.

### 3.1. Midline Approach

The conventional midline approach remains the historical standard for coccygectomy treatment. This technique involves a direct linear incision over the midline of the lower sacrum and coccyx, providing straightforward access to the surgical area. While offering excellent exposure and familiar anatomical landmarks, the midline approach carries an increased risk of wound complications compared to alternative methods. These complications primarily stem from the incision’s location within the natal cleft, an environment prone to moisture retention and bacterial colonization [10].

The surgical technique follows a systematic progression: careful dissection through subcutaneous tissue leads to identification of the sacrococcygeal joint or intercoccygeal joints, depending on the planned resection level. Following exposure, the coccyx is methodically detached from surrounding ligamentous and muscular attachments. Meticulous hemostasis and layered wound closure complete the procedure. This approach typically serves patients with traumatic coccygodynia or those who have failed conservative management [11].

### 3.2. Paramedian Approach

The paramedian approach represents an evolution in surgical techniques, developed specifically to address the wound healing challenges associated with midline incisions. Wound healing complications following coccygectomy can be stratified into three distinct categories that have been reported in the literature [2,12]. Superficial complications include minor wound dehiscence, delayed epithelialization, and superficial infection. Deep wound complications, characterized by complete dehiscence, tissue necrosis, or deep infection, require more aggressive intervention including surgical debridement and culture-directed antibiotics. The most severe category involves deep wound complications with systemic inflammation, presenting with abscess formation, osteomyelitis, or systemic inflammatory response. Prevention remains paramount, with careful attention to surgical techniques, appropriate wound closure, and postoperative wound care protocols. The paramedian method, documented primarily through technical papers and institutional studies, positions the incision laterally offset from the midline, typically on either side of the natal cleft. By avoiding the midline’s inherently problematic environment, this approach has demonstrated superior outcomes in infection prevention and wound healing [13,14].

Technically, the paramedian approach requires creating an incision parallel and adjacent to the sacrococcygeal junction. While following similar dissection principles as the midline approach, this technique demands heightened attention during tissue manipulation due to the altered approach angle and proximity to critical structures such as the rectum. The modified access path necessitates careful soft tissue handling and precise anatomical orientation [13,14].

This approach has proven particularly valuable for specific patient populations, notably those with obesity or deep natal clefts, where traditional midline access poses elevated complication risks. The technique’s ability to provide a more favorable wound healing environment makes it an increasingly preferred option, especially for complex cases where wound healing concerns are paramount [14].

## 4. Extent of Resection

### 4.1. Partial Coccygeal Resection

Partial coccygeal resection involves selective removal of coccygeal segments, typically focusing on the distal portion. This technique is particularly indicated when pathology localizes to specific segments, such as isolated fractures or focal degenerative changes in the lower coccygeal joints. The selective nature of partial resection offers potential advantages including reduced surgical trauma, shorter recovery periods, and decreased complication risks [15,16]. However, outcome data presents a mixed picture. While some studies report excellent pain relief with minimal complications, others document higher symptom recurrence rates compared to complete resection. Patient selection proves crucial, requiring thorough preoperative assessment including detailed pain characterization and comprehensive imaging evaluation [17].

### 4.2. Complete Coccygeal Resection

Complete coccygectomy, involving total excision of the coccyx, typically serves as the primary approach for diffuse coccygeal pathology or after failed partial resection. This technique demonstrates efficacy in cases with widespread coccygeal involvement or complex pain patterns [18]. The procedure, while more extensive than partial resection, often provides definitive treatment for refractory cases. However, surgeons must carefully weigh its benefits against increased technical demands and potential complications. These include greater blood loss, extended recovery times, and elevated risks of wound complications. Despite these challenges, the literature consistently reports high patient satisfaction rates and significant pain improvement following complete resection [17].

### 4.3. Comparative Outcomes of Extent of Resection

The current literature demonstrates no clear consensus on superiority between partial versus complete resection for pain control outcomes [19,20]. Treatment selection requires comprehensive evaluation of multiple clinical factors. These include underlying pathology extent, anatomical considerations, overall patient health status plus specific therapeutic goals. Success rates correlate strongly with meticulous patient selection through detailed preoperative assessment including advanced imaging, physical examination findings and thorough pain characterization.

## 5. Wound Closure Techniques

### 5.1. Off-Center Closure

The off-center closure technique involves making an incision slightly lateral to the midline, akin to the paramedian approach in coccygectomy. By positioning the wound away from the natal cleft—a region prone to moisture and bacterial colonization—this method aims to reduce the risk of wound dehiscence and infection commonly associated with midline incisions [21]. It is particularly beneficial for patients with a deep natal cleft or higher infection risk, such as those who are obese or have poor wound healing capacity.

### 5.2. Z Plasty

Z Plasty is a surgical method used to reorient wound tension lines, making it valuable in areas susceptible to tightness and scarring. In coccygectomy, applying Z Plasty can redistribute tension along the wound, thereby decreasing the risk of scar contracture and dehiscence. The technique involves creating angled flaps in a ‘Z’ shape; when closed, these flaps alter the scar’s direction, potentially improving both functional and cosmetic outcomes. This method is considered when there is a significant risk of wound healing complications or in revision surgeries where scar tissue is a concern [10].

### 5.3. Paramedian Curvilinear Skin Incision

This technique combines the advantages of an off-midline approach with a curvilinear incision to minimize wound tension. By following the natural contours of the sacral area, the curvilinear incision promotes a more natural healing process and reduces complications. Avoiding the high-tension and high-moisture midline area, this method effectively decreases wound dehiscence and infection rates [13,14].

Each of these wound closure techniques offers distinct advantages in postoperative recovery for coccygectomy patients. The choice depends on the patient’s anatomy, surgical extent, and the surgeon’s experience. The primary goal is to ensure optimal healing, minimize complications, and enhance overall surgical outcomes [10,13,21].

### 5.4. Minimally Invasive: The New Frontier

Coccygeoplasty is a relatively new, minimally invasive procedure designed for patients with coccyx hypermobility, where excessive coccyx movement leads to chronic pain [20]. Serving as an alternative to more invasive surgeries like coccygectomy [20], coccygeoplasty involves the percutaneous injection of bone cement (polymethylmethacrylate) into the coccyx under fluoroscopic guidance [22,23]. This stabilizes the coccygeal joints, reducing mobility and alleviating pain associated with movement.

Advantages of coccygeoplasty include its minimally invasive nature, shorter recovery times, and reduced complication risks compared to traditional surgical interventions. Patients often experience immediate pain relief following the procedure. However, potential risks such as infection, cement leakage, and allergic reactions must be considered.

### 5.5. Minimally Invasive Endoscopic Approach

The Minimally Invasive Endoscopic Approach represents an evolution in the surgical treatment of coccygodynia, focusing on reducing tissue trauma and enhancing recovery. This technique involves the use of endoscopic tools to perform coccygectomy or coccygeal joint injections [24,25].

**Figure 1:**
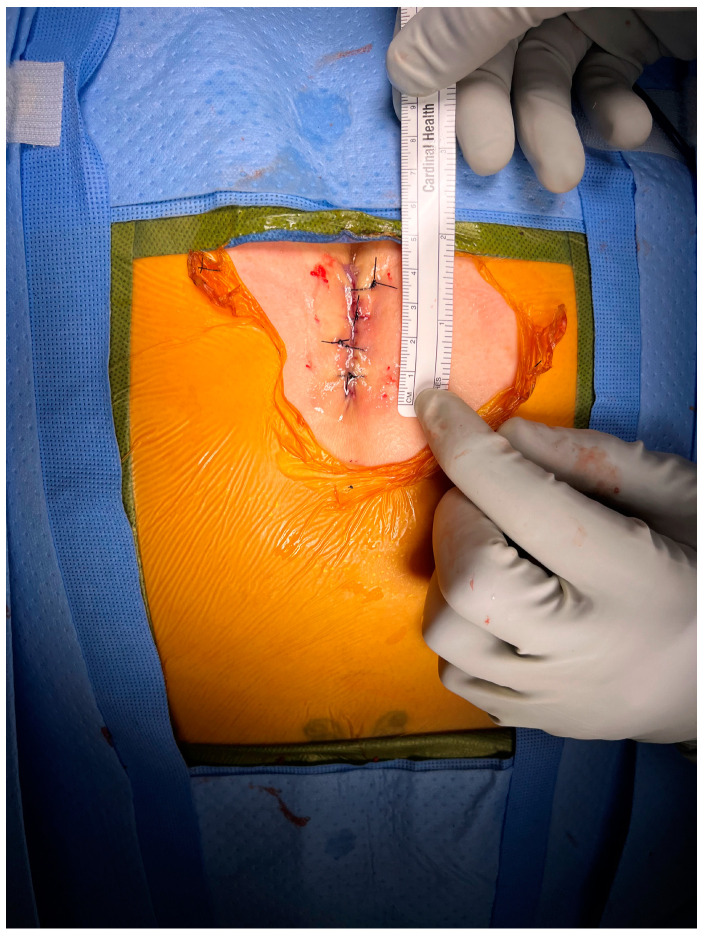
Midline wound closure after a minimally invasive coccygetomy.

**Figure 2:**
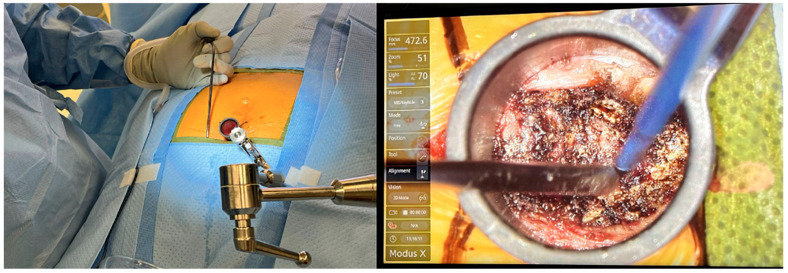
Minimally Invasive Coccygectomy Performed through METRx tube. The extent of exposure achieved during endoscopic approach can be visualized, as indicated by surgical instruments.

During an endoscopic coccygectomy, small incisions are made, and endoscopic instruments are inserted to visualize and operate on the coccyx (Figure 1 and Figure 2). This approach allows for precise removal of the affected coccyx segment while minimizing damage to surrounding tissues. The endoscopic method provides surgeons with better visibility, increased accuracy, and the ability to perform the surgery with less invasive maneuvers [24].

These new approaches carry significant clinical implications, including the potential for lower wound complication rates and accelerated recovery times. Patients may also benefit from smaller scars and less postoperative care. However, this technique requires specialized equipment and training, and its long-term outcomes are still being evaluated in ongoing clinical studies [24].

## 6. Outcomes

The effectiveness of coccygectomy in managing coccygodynia is well-documented, with outcomes suggesting substantial improvements across various surgical approaches. However, nuances in surgical treatment have evolved over time that merit elucidation. For instance, Negappa et al. demonstrated that use of a curvilinear paramedian skin incision can produce significant enhancements in patient outcomes [13]. Their cohort of 45 patients demonstrated improved Oswestry Disability Index (ODI) and Visual Analogue Scale pain (VAS) scores, with pre- and post-intervention means of 29 vs. 7.7 for ODI and 8 vs. 2 for VAS. Furthermore, post-surgical patient satisfaction scores averaged 8 out of 10, suggesting a good to excellent outcome. These findings underline not only the procedure’s capability to enhance quality of life but also its effectiveness in adult and pediatric demographics with tweaking of established modalities.

Close attention must be paid at each point of the procedure, particularly wound closure, to ensure the best possible outcome for the patient. Here, remarkably, the complication rates observed were either on par with or lower than those reported in previous studies, especially in terms of wound infections, which were seen in approximately 11% of cases [13]. Recent advancements in surgical techniques, such as the implementation of the ‘off-center’ and ‘Z-Plasty’ wound closure method, have potential to significantly influence the landscape of coccygectomy. These innovations have led to a lower infection rate and enhanced long-term clinical outcomes, further establishing coccygectomy as an effective intervention for intractable coccygodynia [10,21].

When evaluating the influence of coccygodynia’s etiology on surgical outcomes, it has been found that patients suffering from traumatic coccygodynia typically experience better functional outcomes post-surgery [10]. This distinction is evident in the marked improvement of scores across SF-36 Mental and Physical Component Summaries, alongside reductions in VAS and ODI scores, suggesting a considerable enhancement in life quality following coccygectomy.

The efficacy of coccygectomy across various etiologies was confirmed by Sagoo et al. through a systematic review and meta-analysis, aggregating data from 21 studies to validate the procedure. Their analysis revealed a consistent surpassing of the minimal clinically important difference (MCID) threshold for pain relief over various follow-up periods. Furthermore, an 8% pooled incidence rate of complications was observed, reinforcing previous reports on the procedure’s safety, particularly regarding surgical site infections and wound dehiscence [21,26].

The rate of post-operative clinical improvement and specifically pain is an important clinical endpoint across each of the various methods used to treat refractory coccygodynia [8]. For instance, one large systematic review and meta-analysis investigating 1980 patients across 64 studies identified a pooled baseline pain score after >12 months of conservative care of 5.00 on a Numerical Rating Scale of pain vs. 2.32 for patients treated with coccygectomy. This is despite surgically treated patients having a preoperative pain score nearly one point higher than conservatively treated patients, 7.54 vs. 6.69. This effect was replicated by a second systematic review with a partially overlapping evidence window, wherein Sagoo et al. noted a mean reduction in pain score of approximately five points across all included studies [17]. This effect appears to be conserved across several levels of resection, with both total and partial coccygectomy predicting significant pain relief in treatment-resistant coccydynia [19,27].

**Figure 3:**
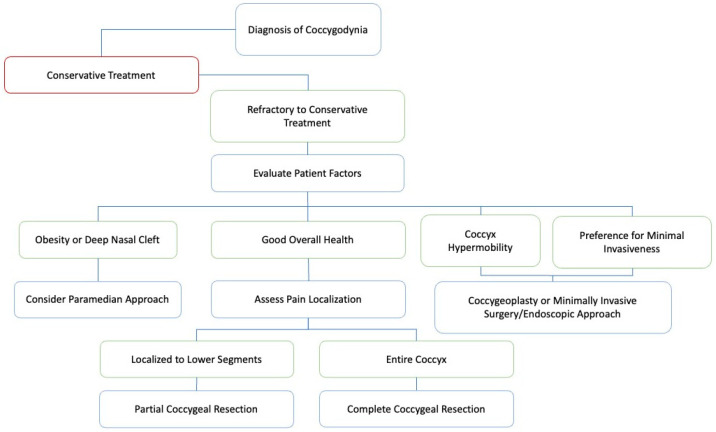
Coccygodynia: A Decision-Making Algorithm. Key decision-making steps in the surgical management of refractory coccygodynia are outlined, including indications for conservative treatment, paramedian, endoscopic, coccygeoplasty, partial resection, and complete resection.

These findings clarify the highly individualized role of coccygectomy as a treatment modality for refractory coccygodynia, one capable of producing marked improvements in pain relief, disability outcomes, and patient satisfaction. We proposed a potential decision-making algorithm that accounts for multiple factors discussed in this comprehensive review (Figure 3). The careful selection of surgical techniques, coupled with a detailed consideration of patient-specific factors such as etiology and existing conditions, is paramount in optimizing surgical outcomes and setting realistic expectations for postoperative recovery.

## 7. Limitations

Several limitations merit consideration in this review. Significant heterogeneity in outcome measures across studies, including inconsistent use of validated tools and varying follow-up periods, complicates direct comparison between surgical techniques. Newer minimally invasive approaches, while promising, lack long-term follow-up data to fully evaluate their durability and complication profiles. Furthermore, variation in surgical technique descriptions across studies impacts procedure reproducibility, particularly for newer modifications. Publication bias may also influence available evidence, especially for novel techniques where successful outcomes are more likely to be reported. Despite these limitations, the current evidence supports coccygectomy’s efficacy in properly selected patients, with technique selection individualized to patient factors and surgeon experience. Future research with standardized outcome measures and longer follow-up periods would strengthen the evidence base for surgical decision-making.

## 8. Conclusions

This review highlights the critical importance of individualized surgical approaches in treating coccygodynia. Balancing traditional techniques like midline and paramedian incisions with innovative methods such as coccygeoplasty and endoscopic procedures is essential. The choice between partial and complete resection, along with the use of specialized wound closure techniques—such as off-center closure, Z Plasty, and paramedian curvilinear incisions—is pivotal in optimizing patient outcomes. Emerging minimally invasive techniques, particularly coccygeoplasty and endoscopic coccygectomy, show significant promise in reducing recovery times and complication rates. Surgeons must carefully consider these diverse options in light of each patient’s unique clinical background to tailor surgical interventions and achieve the best possible results.

## Figures and Tables

**Table 1:** Overview of surgical techniques for coccygectomy.

Technique	Indications	Procedure Summary	Key Advantages	Key Disadvantages	Typical Outcomes
Midline Approach	Traumatic Coccygodynia or Failed Conservative Management	Incision over Lower Sacrum and Coccyx followed by Detachment of Coccyx	Direct Access, Standard Technique	Higher Risk of Wound Complications	High Rate of Pain Relief, Standard Recovery
Paramedian Approach	Deep Natal Cleft, Obese Patients	Off-Midline Incision, Similar Detachment as Midline	Reduced Wound Complications	More Cautious Dissection Required	Reduced Infection Rates, Quicker Healing
Partial Coccygeal Resection	Localized Pain to Lower Coccyx	Removal of Distal Coccyx Segments	Less Invasive, Shorter Recovery	Higher Recurrence of Symptoms	Satisfactory Pain Relief, Minimal Complications
Complete Coccygeal Resection	Diffuse Pain, Entire Coccyx Involved	Removal of Entire Coccyx	Effective in Eliminating Pain	Greater Blood Loss, Longer Recovery	High Success Rates, Satisfactory Pain Relief
Coccygeoplasty	Coccyx Hypermobility	Percutaneous Injection of Bone Cement into Coccyx	Minimally Invasive, Immediate Pain Relief	Risks of Infection, Cement Leakage	Immediate Pain Relief, Short Recovery
Endoscopic Approach	Preference for Minimally Invasive	Small Incisions, Endoscopic Tools for Coccyx Removal	Less Tissue Trauma, Faster Recovery	Requires Specialized Equipment and Training	Reduced Postoperative Pain, Faster Recovery

## Data Availability

No new data were created or analyzed in this study. Data sharing is not applicable to this article.
